# Ibogaine Acute Administration in Rats Promotes Wakefulness, Long-Lasting REM Sleep Suppression, and a Distinctive Motor Profile

**DOI:** 10.3389/fphar.2018.00374

**Published:** 2018-04-27

**Authors:** Joaquín González, José P. Prieto, Paola Rodríguez, Matías Cavelli, Luciana Benedetto, Alejandra Mondino, Mariana Pazos, Gustavo Seoane, Ignacio Carrera, Cecilia Scorza, Pablo Torterolo

**Affiliations:** ^1^Laboratorio de Neurobiología del Sueño, Departamento de Fisiología, Facultad de Medicina, Universidad de la República, Montevideo, Uruguay; ^2^Departamento de Neurofarmacología Experimental, Instituto de Investigaciones Biológicas Clemente Estable, Montevideo, Uruguay; ^3^Laboratorio de Síntesis Orgánica, Departamento de Química Orgánica, Facultad de Química, Universidad de la República, Montevideo, Uruguay

**Keywords:** REM sleep, wakefulness, ibogaine, psychedelics, hallucinogens

## Abstract

Ibogaine is a potent psychedelic alkaloid that has been the focus of intense research because of its intriguing anti-addictive properties. According to anecdotic reports, ibogaine has been originally classified as an oneirogenic psychedelic; i.e., induces a dream-like cognitive activity while awake. However, the effects of ibogaine administration on wakefulness (W) and sleep have not been thoroughly assessed. The main aim of our study was to characterize the acute effects of ibogaine administration on W and sleep. For this purpose, polysomnographic recordings on chronically prepared rats were performed in the light phase during 6 h. Animals were treated with ibogaine (20 and 40 mg/kg) or vehicle, immediately before the beginning of the recordings. Furthermore, in order to evaluate associated motor behaviors during the W period, a different group of animals was tested for 2 h after ibogaine treatment on an open field with video-tracking software. Compared to control, animals treated with ibogaine showed an increase in time spent in W. This effect was accompanied by a decrease in slow wave sleep (SWS) and rapid-eye movements (REM) sleep time. REM sleep latency was significantly increased in animals treated with the higher ibogaine dose. While the effects on W and SWS were observed during the first 2 h of recordings, the decrement in REM sleep time was observed throughout the recording time. Accordingly, ibogaine treatment with the lower dose promoted an increase on locomotion, while tremor and flat body posture were observed only with the higher dose in a time-dependent manner. In contrast, head shake response, a behavior which has been associated in rats with the 5HT_2A_ receptor activation by hallucinogens, was not modified. We conclude that ibogaine promotes a waking state that is accompanied by a robust and long-lasting REM sleep suppression. In addition, it produces a dose-dependent unusual motor profile along with other serotonin-related behaviors. Since ibogaine is metabolized to produce noribogaine, further experiments are needed to elucidate if the metabolite and/or the parent drug produced these effects.

## Introduction

Ibogaine is a natural occurring indole alkaloid found in the root bark of the shrub *Tabernanthe iboga*, originally from Congo and Gabon, as well as in other plants of the Apocynaceae family (Lavaud and Massiot, 2017). It has become widely known in the western world because of its claimed properties to reduce addiction to drugs of abuse and craving (Alper et al., 1999). These effects have been confirmed in pre-clinical studies in rodents, where ibogaine decreases self-administration of a variety of drugs such as opioids (Lotsof, 1995), cocaine (Cappendijk and Dzoljic, 1993), alcohol (Rezvani et al., 1995), and nicotine (Glick et al., 1998). In humans, the reduction of addiction and craving by ibogaine has been highlighted in many anecdotal reports and observational studies (Schenberg et al., 2014; Brown and Alper, 2017).

Ibogaine has been classified as an oneirogenic psychedelic drug for its ability to induce vivid dream-like episodes while awake with eyes closed, without loss of contact with the environment (Naranjo, 1973). This dream-like activity does not produce the typical interferences in thinking, identity distortions, and space–time alteration produced by the traditional psychedelics drugs (also known as hallucinogens) such as lysergic acid diethylamide (LSD), mescaline, and dimethyltryptamine (DMT), which are pharmacologically classified as 5-HT_2A_ receptor agonists (Naranjo, 1973; Glennon, 1990; Lotsof, 1995; Nichols, 2016). However, the effect of ibogaine on wakefulness (W) and sleep remains unclear, existing only a few early studies regarding this issue. An early report in cats described that the administration of ibogaine (2–10 mg/kg doses) produced an activation of the electroencephalogram (EEG) that resembles the effect of the electrical stimulation of the activating reticular system (Schneider and Sigg, 1957). This effect was reduced in the classical “decerebrate” animal model, suggesting that the reticular formation plays a key role in the ibogaine effects. Moreover, they demonstrated that atropine, a muscarinic cholinergic antagonist, blocked the EEG-activating effect induced by ibogaine. Because of this result, the authors suggested that ibogaine effect depends on the activation of the cholinergic system. Da Costa et al. (1980), Da Costa-Rochette et al. (1981) analyzed the effects of others iboga alkaloids on chronically implanted cats. They found that tabernanthine tartrate and tabernanthine *p*-chlorophenoxyacetate provoked an increase in W and a reduction of both slow wave sleep (SWS) as well as rapid-eye movements (REM) sleep. However, the opposite effect was found when other tabernanthine derivatives (claimed as methoxy-16 ibogaine tartrate and methoxy-16 tabernanthine tartrate) were administered (Da Costa et al., 1980; Da Costa-Rochette et al., 1981).

When considering the behavioral responses induced by ibogaine in animal models, different effects have been reported depending on the dose, time points assayed, and length of the recordings. In one of the early reports in cats, it has been described that ibogaine administration (2–10 mg/kg) immediately produced an unusual excitatory effect that evolved into reactions of rage and fear (Schneider and Sigg, 1957). A more recent study in rats showed that ibogaine (1 and 10 mg/kg, i.v.) promoted a dose-dependent increase in locomotor activity during 30 min after administration (Baumann et al., 2001a). For higher doses [10–40 mg/kg, intraperitoneal (i.p.)], ibogaine produced deleterious effects in the vestibular function and a dose-dependent reduction in the detection of sensory stimuli in rats (Kesner, 1995). In addition, rats treated with 30 and 40 mg/kg were very inactive and appeared to be in a state of “suspension” (Kesner, 1995). These facts resemble subjective reports in humans, where ibogaine (4–5 mg/kg) promotes the desire to lie down because of a loss of equilibrium while trying to walk and directs the attention inwards (Naranjo, 2016). Previous studies also demonstrated that during the first 3–4 h after administration in animals, ibogaine (30–40 mg/kg or higher doses) produced serotonin syndrome-like behaviors such as tremor, piloerection, flat body posture, and forepaw tapping (Schneider and Sigg, 1957; Glick et al., 1994; Pearl et al., 1997; Baumann et al., 2001a; Haberzettl et al., 2013). It is well known that hallucinogens induce paroxysmal rotational movement of the head in rodents, that is mediated by 5-HT_2A_ receptor activation (Halberstadt et al., 2011; Nichols, 2016). This behavior is called head twitch response in mice and head shake response (HSR) in rats (Corne and Pickering, 1967; Yamamoto and Ueki, 1975; Bedard and Pycock, 1977; Halberstadt et al., 2011). To the best of our knowledge, no previous studies explored if ibogaine promotes this behavior in rodents.

The main aim of the present study was to characterize ibogaine effects on sleep and W using rat as a model system. For this purpose, we performed polysomnographic recordings in chronically prepared rats and studied the acute effects of ibogaine, at doses that have been previously employed in drug self-administration studies (20 and 40 mg/kg). In addition, in order to analyze the animal behavior during the waking state, we characterized the motor effects induced by the same doses of ibogaine in naive animals, using an open-field (OF) assay.

## Materials and Methods

### Ibogaine

Total iboga alkaloid extract from the root bark of *T. iboga* was obtained from IbogaWorld and purified as follows. The material was suspended in aqueous 10% NaOH solution, which was extracted with ethyl acetate (4 × 200 ml). The combined organic layers were dried with Na_2_SO_4_ and evaporated *in vacuo*. The residue was purified by column chromatography (SiO_2_, CH_2_Cl_2_:MeOH 9:1 + 0.1% NH_4_OH). The obtained free base was further crystallized from ethanol. Ibogaine HCl was prepared dissolving the free base in dried acetone under Argon atmosphere and the equivalent amount of HCl (aq, 36%) was added. Ibogaine hydrochloride was filtered, washed with cold acetone, dried under vacuum, and characterized by ^1^H- and ^13^C-NMR (see Supplementary Materials for details). Purity was determined as 96.4% by GC–MS (see Supplementary Materials for details). Dissolution of ibogaine-HCl to prepare the samples for i.p. injection was carried out using warm saline that was previously degassed by nitrogen bubbling.

### Experimental Animals

Wistar adult rats were maintained on a 12-h light/dark cycle (lights on at 07.00 h) and housed four–six per cage before behavioral testing. Food and water were freely available. Twenty-six animals (270–300 g) were used for all performed studies: eight animals were used for sleep recordings and 18 rats were used for the evaluation of the motor activity. The animals were determined to be in good health by veterinarians of the institution. All experimental procedures were conducted in agreement with the National Animal Care Law (No. 18611) and with the “Guide to the care and use of laboratory animals” (8th edition, National Academy Press, Washington DC, 2010). Furthermore, the Institutional Animal Care Committee approved the experimental procedures. Adequate measures were taken to minimize pain, discomfort, or stress of the animals, and all efforts were made to use the minimal number of animals necessary to obtain reliable scientific data.

### Surgical Procedures

Eight animals selected for sleep experiments were chronically implanted with electrodes to monitor the states of sleep and W. We employed similar surgical procedures as in our previous studies (Benedetto et al., 2013; Cavelli et al., 2015, 2017b). Anesthesia was induced with a mixture of ketamine–xylazine (90 mg/kg; 5 mg/kg i.p., respectively). The rat was positioned in a stereotaxic frame and the skull was exposed. To record the EEG, stainless steel screw electrodes were placed on the skull above frontal, parietal, occipital cortices (bilateral), the right olfactory bulb, and cerebellum (reference electrode).

To record the electromyogram (EMG), two electrodes were inserted into the neck muscle. The electrodes were soldered into a 12-pin socket and fixed onto the skull with acrylic cement. At the end of the surgical procedures, an analgesic (Ketoprofen, 1 mg/kg, s.c.) was administered. After the animals had recovered from the preceding surgical procedures, they were adapted to the recording chamber for 1 week.

### Experimental Sessions

#### Sleep Recordings

Animals were housed individually in transparent cages (40 × 30 × 20 cm) containing wood shaving material in a temperature-controlled (21–24°C) room, with water and food *ad libitum*. Experimental sessions were conducted during the light period, between 10 AM and 4 PM in a sound-attenuated chamber with Faraday shield. The recordings were performed through a rotating connector, to allow the rats to move freely within the recording box.

Polysomnographic data were acquired and stored in a computer for further analysis using Spike 2 software (CED, Cambridge, United Kingdom). The states of sleep and W were determined in 10 s epochs. W was defined as low voltage fast waves in frontal cortex, a mixed theta rhythm (4–7 Hz) in occipital cortex, and relatively high EMG activity. Light sleep (LS) as high voltage slow cortical waves interrupted by low voltage fast EEG activity. SWS was defined as continuous high amplitude slow waves and sleep spindles in frontal, parietal, and occipital cortices associated with a reduced EMG amplitude; while REM sleep as low voltage fast frontal waves, a regular theta rhythm in the occipital cortex, and a silent EMG except for occasional twitches. Total time spent in W, LS, SWS, and REM sleep, as well as the duration and the number of episodes over a 6 h recording period was analyzed. Sleep latencies were also evaluated. Besides, the time spent in each state was analyzed separately in blocks of 2 h (0–2, 2–4, and 4–6 h) (Monti et al., 2015).

In order to study the effect of ibogaine on sleep and W, at the beginning of the recordings each rat received ibogaine 20 mg/kg (*I*_20_), 40 mg/kg (*I*_40_), or vehicle (saline) i.p., in different days in a counterbalance order; the wash-out period between doses was 3 days. These doses have been extensively used in preclinical addiction studies (Glick et al., 1991, 1992; Cappendijk and Dzoljic, 1993).

#### Motor Behavior

Eighteen naive (not operated) rats were used in these experiments. Animals were brought to the experimental room in their home cages, identified, and weighed prior to the behavioral test. An OF apparatus consisting of a square area (45 cm wide × 45 cm long × 40 cm high) with transparent plastic walls indirectly illuminated (35 luxes) to avoid reflection and shadows were employed. The OF was placed in a quiet experimental room with controlled temperature (22 ± 2°C). As rats were not habituated to the OF before drug or vehicle administration, novelty-induced motor activity was automatically recorded by a camera connected to a computer equipped with the Ethovision XT 12.0 software (Noldus, Netherlands) located above the OF. Using this video tracking software, we specifically measured the total distance traveled in meters (m) during 120 min (5 min bin), starting immediately after the drug or vehicle administration. Animals were randomly assigned to different experimental groups, where each animal received *I*_20_, *I*_40_, or saline (*n* = 6 per group), and were used only once. Specific behaviors were assessed by a trained investigator every 30 min during 2 h after ibogaine administration. Each evaluation session lasted 5 min. The number of rearings was taken as an index of the vertical exploratory behavior to evaluate the animal habituation to the environment. Serotonin syndrome-like continuous behaviors such as tremor, flat body posture, piloerection, hind limb abduction, and Straub tail (Haberzettl et al., 2013) were scored using a graded scale: 0, absent; 1, equivocal; 2, present; and 3, intense (Spanos and Yamamoto, 1989; Reyes-Parada et al., 1996; Baumann et al., 2001b). In the case of forepaw treading (an intermittent behavior), an additive score of 1 was given every time the animal displayed this conduct. However, to allow a comparison with the continuous behaviors, a score of 5 (five times the animal displayed a FPT behavior) was considered as intense (3, in the abovementioned scale). Finally, the number of HSR defined as short and firm movement of the head in any direction was also recorded (Haberzettl et al., 2014).

During all experiments, the OF was cleaned with alcohol 30% before placing the following rat. All experiments were done between 9 AM and 3 PM.

### Data Analysis

#### Sleep Recordings

All values are presented as mean ± SEM. The experimental design for the sleep analysis was a within-subject design, where statistical significance of the differences among groups (ibogaine 0, 20, and 40 mg/kg) was evaluated utilizing one-way repeated measures analysis of variance (ANOVA) and Bonferroni as a *post hoc* test (Monti et al., 2015). When sphericity criteria were not accomplished (tested by Mauchly’s test), the Greenhouse–Geisser correction was applied. Statistical significance was set at *p* < 0.05.

#### Motor Behavior

Depending on the comparison performed, data from motor activity were analyzed by two-way (treatment, time, and interaction between factors) ANOVA for repeated measures followed by Newman–Keuls multiple comparison *post hoc* test; or by one-way (treatment) ANOVA for independent measures followed by Newman–Keuls multiple comparison test. In all cases, statistical significance was set at *p* < 0.05.

## Results

### Ibogaine’s Effect on Wakefulness and Sleep

**FIGURE 1:**
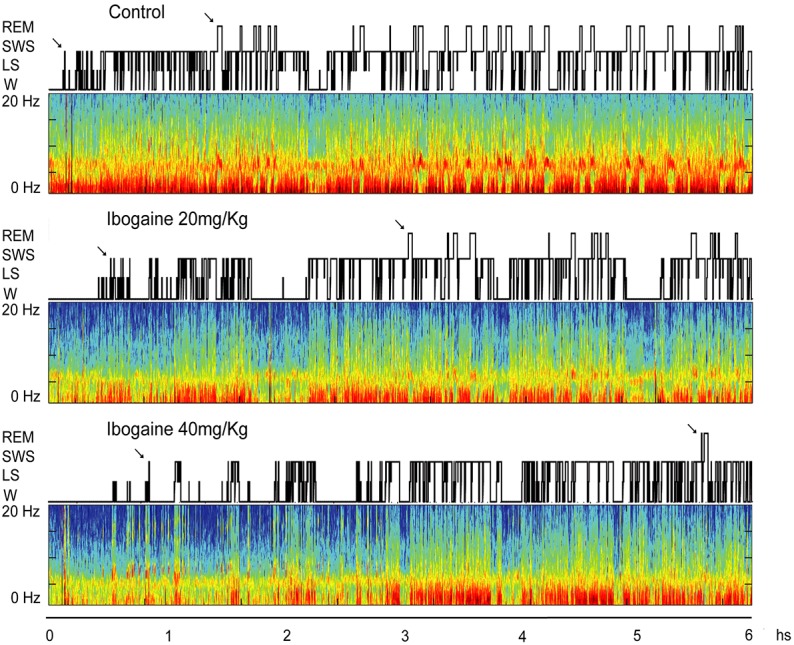
Hypnograms and spectrograms (0.1–20 Hz) from the parietal cortical recordings of a representative animal are shown after saline, ibogaine 20 and 40 mg/kg. Arrows in the hypnogram indicate SWS and REM sleep latencies. During wakefulness (W) and REM sleep, theta activity (4–9 Hz) in the spectrograms can be readily observed. During SWS sleep, delta activity (0.5–4 Hz) is more prominent and there are intermittent episodes of sigma activity (9–15 Hz), which correspond to the presence of sleep spindles. Color calibration of the spectrogram is not exhibited (larger power is exhibited in red). Ibogaine increased W and reduced REM sleep time. LS, light sleep; REM, rapid eyes movements sleep; SWS, slow wave sleep.

**Figure 1** shows a typical hypnogram and spectrogram of a representative animal following saline, *I*_20_, and *I*_40_ administration. Compared to control, *I*_20_ [*F*_(1.1,8.3)_ = 10.7, *p* < 0.01] and *I*_40_ [*F*_(1.1,8.3)_ = 10.7, *p* < 0.05] increased the time spent in W (**Figure 1** and **Table 1**). This effect was accompanied by a decrease in SWS time, *I*_20_ [*F*_(2,14)_ = 14.7, *p* < 0.01], *I*_40_ [*F*_(2,14)_ = 14.7, *p* = 0.01]. In addition, the total amount of REM sleep was diminished in animals treated with *I*_20_ [*F*_(2,14)_ = 19.3, *p* < 0.01] and *I*_40_ [*F*_(2,14)_ = 19.3, *p* < 0.005]. No differences on the total amount of LS were observed.

**Table 1:** Effects of intraperitoneal injections of ibogaine on sleep and waking parameters during total recording time.

	Control	Ibogaine 20 mg/kg	Ibogaine 40 mg/kg
**Wakefulness**			
Total duration (min)	95.1 ± 7.8	135.5 ± 9.8*	182.6 ± 26.6*
Number of episodes	118.0 ± 8.9	130.8 ± 8.1	127.1 ± 10.4
Episodes duration (min)	0.8 ± 0.0	1.0 ± 0.1*	1.5 ± 0.2
**Light sleep (LS)**			
Total duration (min)	32.6 ± 2.6	39.4 ± 3.0	37.6 ± 4.3
Number of episodes	152.6 ± 3.4	173.7 ± 4.4	163.2 ± 5.4
Episodes duration (min)	0.21 ± 0.0	0.2 ± 0.0	0.22 ± 0.0
**Slow wave sleep (SWS)**			
Total duration (min)	197.8 ± 8.0	162.3 ± 7.3*	127.4 ± 19.6*
Number of episodes	141.5 ± 7.4	152.5 ± 13.7	124.8 ± 7.8
Episodes duration (min)	1.4 ± 0.9	1.1 ± 0.8	1.0 ± 0.8*
Latency	9.1 ± 1.7	21.8 ± 3.6	53.2 ± 14.9
**REM sleep**			
Total duration (min)	33.6 ± 2.5	22.0 ± 2.8*	11.5 ± 3.7*
Number of episodes	25.1 ± 2.3	19.6 ± 2.6*	11.8 ± 4.7*
Episodes duration (min)	1.3 ± 0.0	1.1 ± 0.1	0.8 ± 0.2
Latency (min)	72.5 ± 2.7	137.1 ± 24.8	229.8 ± 43.4*

*Data are presented as mean ± standard error of eight rats. ^∗^Symbols denote significant difference compared with control values using ANOVA repeated measures followed by Bonferroni test (p < 0.05).*

When considering the duration and number of episodes (**Table 1**), we found that compared to control, there was a significant increase in the duration of the individual W episodes after *I*_20_ [*F*_(1.1,7.9)_ = 6.4, *p* < 0.05], and a decrease in the duration of SWS episodes for *I*_40_ [*F*_(2,14)_ = 9.5, *p* < 0.005]. Regarding REM sleep, both doses of ibogaine reduced the total number of episodes [*F*_(1.2,8.5)_ = 10.5; *p* < 0.05 for *I*_20_ and *I*_40_] without affecting the episodes’ duration. Finally, REM sleep latency increased following *I*_40_ administration [*F*_(2,14)_ = 9.6, *p* < 0.05] (**Table 1**), while the latency to LS and SWS was not affected.

**FIGURE 2:**
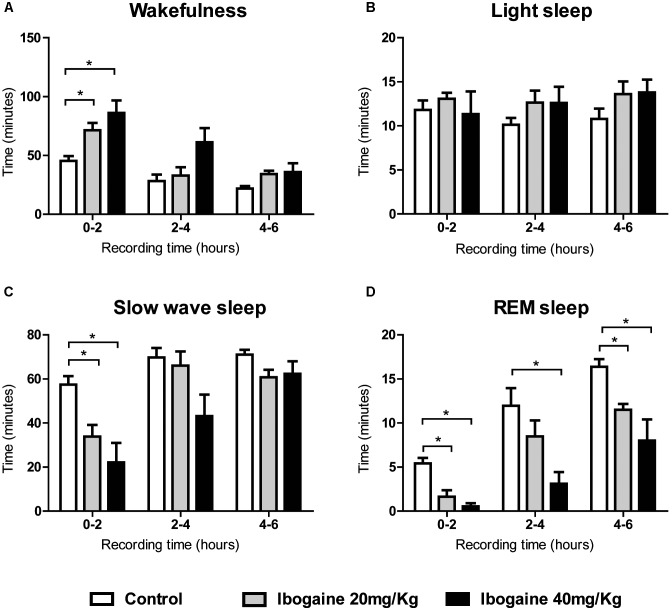
Effects of ibogaine administration on sleep and W. Graphic chart shows the mean ± SEM time spent in **(A)** W, **(B)** light sleep, **(C)** slow wave sleep, and **(D)** REM sleep after administration of saline and ibogaine (20 and 40 mg/kg), analyzed in 2-h blocks. Group mean differences were determined by one-way ANOVA repeated measures and Bonferroni as *post hoc* test; significant differences (*p* < 0.05) between groups are indicated by asterisks. Eight animals were employed in this analysis.

Ibogaine effects were also analyzed in 2-h blocks (**Figure 2**). Compared to control, the time of W was significantly increased for both doses in the first 2 h of the recording [*F*_(2,14)_ = 9.1, *p* < 0.05 for *I*_20_ and *I*_40_]. This increment was accompanied by a decrease in SWS [*F*_(2,14)_ = 10.2, *p* < 0.05 for *I*_20_, and *p* < 0.01 for *I*_40_] and REM sleep [*F*_(2,14)_ = 17.8, *p* < 0.05 for *I*_20_, and *p* = 0.001 for *I*_40_] without any appreciable change in LS. Within the second 2 h, a decrease in the REM sleep was observed following *I*_40_ [*F*_(2,14)_ = 8.7, *p* < 0.05], while in the last 2 h, both *I*_20_ and *I*_40_ decreased REM sleep time [*F*_(1.1,7.8)_ = 8.3, *p* < 0.005 for *I*_20_, and *p* < 0.05 for *I*_40_].

### Ibogaine’s Effect on Motor Behavior

**FIGURE 3:**
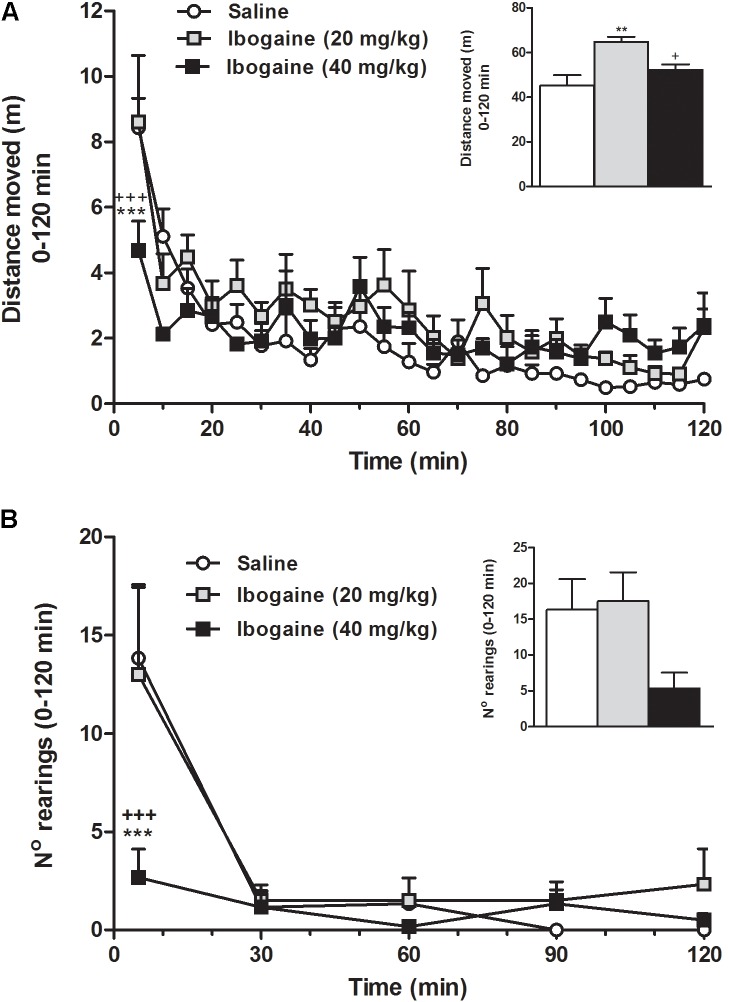
Effect of ibogaine administration (20 and 40 mg/kg) on locomotor activity **(A)** and exploratory activity (**B**; number of rearings) recorded in the OF test during 120 min after ibogaine administration. The inset graphs represent the total locomotor activity and the total number of rearings (0–120 min). Data are expressed as mean ± SEM. The data were analyzed by the two-way ANOVA of repeated measured followed by Newman–Keuls test, and one-way ANOVA of independent measures followed by Newman–Keuls test (insets). ^∗^, respective to saline group; +, respective to ibogaine. ^∗∗∗^, ^+++^*p* < 0.001; ^∗∗^*p* < 0.01; ^+^*p* < 0.05. *N* = 6 per group.

**FIGURE 4:**
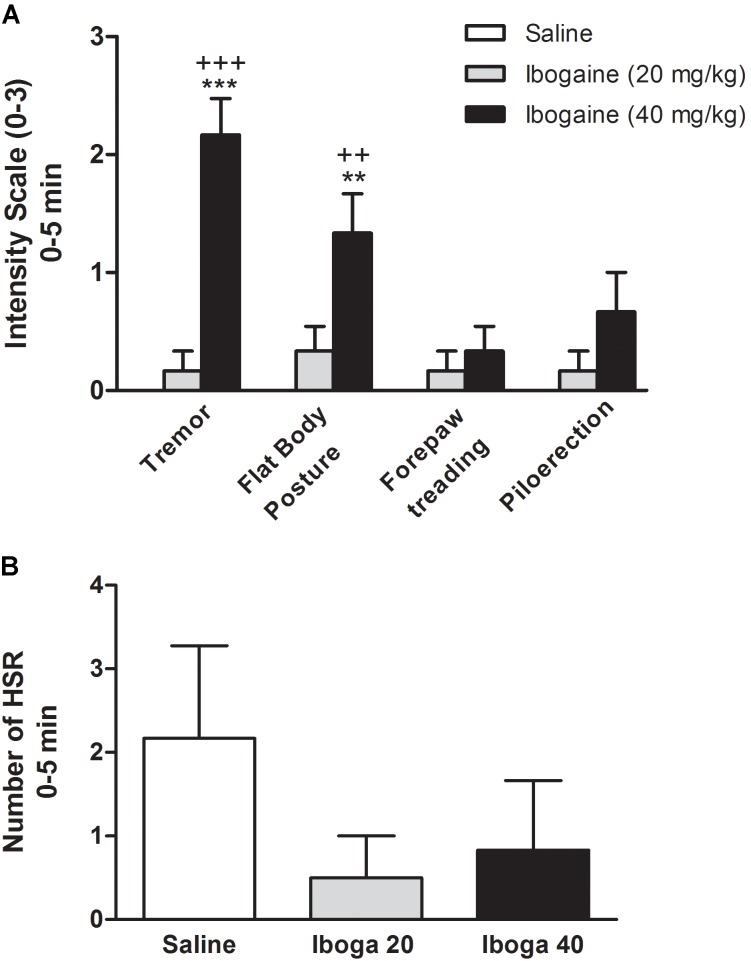
Effects of ibogaine on behavior. Specific behaviors such as tremor, flat body posture forepaw treading, piloerection **(A)**, and head shake response (HSR) **(B)** were shown during the first 5 min after ibogaine administration. In **A**, behaviors were scored using the graded scale: 0, absent; 1, equivocal; 2, present; and 3, intense while in **B**, the number of HSR was scored. Data are expressed as mean ± SEM and analyzed by one-way ANOVA of independent measures followed by Newman–Keuls test. ^∗^, respective to saline group and +, respective to ibogaine. ^∗∗∗^, ^+++^*p* < 0.001; ^∗∗^, ^++^*p* < 0.01. *N* = 6 per group.

In order to obtain further insights about the animal behavior during the ibogaine-induced waking state, a detailed study of motor behaviors was carried out (**Figures 3**, **4**). Compared to the control group, horizontal locomotion was slightly altered by both doses of ibogaine (**Figure 3A**). Two-way ANOVA revealed a significant effect of the treatment [*F*_(2,15)_ = 8.7, *p* < 0.01], time [*F*_(23,345)_ = 12.2, *p* < 0.001], and treatment × time interaction [*F*_(46,345)_ = 1.43, *p* < 0.05]. Newman–Keuls test showed that compared to control and *I*_20_, *I*_40_ elicited a significant decrease (*p* < 0.001) in the distance that the animals moved during the first 5 min; the animals injected with *I*_20_ or saline exhibited a very similar level of locomotor activity (**Figure 3A**). When the total locomotor activity was analyzed (**Figure 3A** inset), one-way ANOVA revealed a significant difference between groups [*F*_(2,15)_ = 8.7, *p* < 0.01]. *Post hoc* analysis showed that *I*_20_ elicited a significant increment in the locomotor activity compared to control (*p* < 0.01) and *I*_40_ (*p* < 0.05) groups. The abovementioned decrease in novelty-induced locomotor activity observed in the *I*_40_-treated animals at the beginning of the recording was not evidenced when the total activity period was considered (**Figure 3A** inset).

Rearing is a component of the natural exploratory behavior directly related to the environment novelty (Fink and Smith, 1980; Geyer et al., 1986; Bardo et al., 1990). In **Figure 3B**, we can observe that naive rats were more active mainly in the first period of the recording session; in fact, the control group seems to be rapidly habituated to the OF apparatus and becoming virtually inactive at the end of the 60-min recording time. Statistical two-way ANOVA analysis revealed a significant effect of the treatment [*F*_(2,15)_ = 4.0, *p* < 0.05], time [*F*_(4,60)_ = 14.6, *p* < 0.001], and the treatment × time interaction [*F*_(8,60)_ = 2.4, *p* < 0.05]. Newman–Keuls test showed that animal injected with *I*_40_ (*p* < 0.001), but not *I*_20_, significantly reduced the ability to induce rearing behavior during the first 5 min, suggesting an alteration in the environment habituation response (**Figure 3B**). When the total rearing activity was considered (**Figure 3B** inset), one-way ANOVA did not reveal a significant difference between groups [*F*_(2,15)_ = 3.4, *p* = 0.06], although this behavior was still not correctly restored.

**Figures 4A,B** show the effect of the ibogaine on the induction of serotonin syndrome-like behaviors and HSR, respectively. In order to simplify, only the first 5 min session is shown. One-way ANOVA analysis revealed significant differences between experimental groups for tremor [*F*_(2,15)_ = 35.9, *p* < 0.0001] and flat body posture [*F*_(2,15)_ = 9.3, *p* < 0.01] but not for forepaw treading [*F*_(2,15)_ = 1.2, *p* = 0.3)] or piloerection [*F*_(2,15)_ = 1.2, *p* = 0.1]. Straub tail and hind limb abduction were not seen (data not shown). *Post hoc* analysis showed that tremor and flat body posture were significantly present immediately after *I*_40_ injection (*p* < 0.001 and *p* < 0.01, respectively, **Figure 4A**), while these behaviors were completely absent at the end of the 60 min session (data not shown). Animal treated with *I*_20_ did not significantly elicit these behavioral signs. Since only these two behaviors were significantly elicited by *I*_40_, an overall serotonin syndrome (Haberzettl et al., 2013) cannot be referred as induced by ibogaine in these conditions. Additionally, one-way ANOVA for the HSR did not reveal significant difference [*F*_(2,15)_ = 1.1, *p* = 0.36] neither for *I*_20_ nor for *I*_40_ during the total 120 min session (**Figure 4B**).

## Discussion

In the present study, we showed that administration of 20 and 40 mg/kg of ibogaine produced a robust effect on sleep and W, promoting a waking state that is accompanied by a robust and long-lasting REM sleep suppressive effect. The higher dose (*I*_40_) showed, in addition, a time-dependent disability to explore a novel environment, as well as disabling behaviors like tremor and flat body posture. It is well-established that ibogaine is rapidly metabolized to its long-lived metabolite noribogaine, so both substances should be taken into account to explain the findings of this study. According to previous reports in rats using i.p. administration (Baumann et al., 2001a), ibogaine concentration in blood rapidly decreases in the first hour while noribogaine concentration is maximum at 2.4 h and lasts up to 24 h. Since it is known that ibogaine induces tremors while noribogaine does not (Baumann et al., 2001a), the fact that we only found tremors during the first hour of recordings for *I*_40_ seems to be in accordance with this pharmacokinetic profile.

Ibogaine administration promoted W; this effect was accompanied by a decrease in the total amount of SWS and REM sleep. While the effects on W and SWS were observed only in first 2 h, the effects on REM sleep lasted through the entire recording. These results may resemble observational studies in humans where ibogaine administration in multiple doses produced difficulties in sleep onset and maintenance immediately after each intake (Wilkins et al., 2017). Ibogaine’s W-promoting effect, in addition, is in accordance with a previous report in cats (Schneider and Sigg, 1957), suggesting that ibogaine induces W in rats, cats, and probably in humans.

Interestingly, a similar impact upon sleep architecture has been reported for traditional psychedelics (5HT_2A_ agonists), such as LSD (also a 5HT_2C_ receptor agonist) and 2,5-dimethoxy-4-iodoamphetamine (DOI, also a 5HT_2C_ agonist) (Depoortere and Loew, 1971; Monti and Jantos, 2006). Chronic LSD treatment in rats promotes an increase in W and a reduction in SWS and REM sleep. Similar to ibogaine, the W-promoting effect was more prominent during the first hour, while it disappeared after 4 h. The authors also found qualitative changes in REM sleep, reporting an increase in phasic episodes as well as an increase in rapid eye movements. Subcutaneous administration of DOI also increases W and decrease SWS and REM sleep. Together these results suggest that ibogaine and traditional psychedelics induce similar effects on sleep architecture. Regarding selective antagonists of 5HT_2A_ and 5HT_2C_ receptors, it was found that their administration promotes SWS; while surprisingly decreases REM sleep time (Monti and Jantos, 2006; Monti et al., 2018). These unexpected results have not been explained yet.

Since dreams are the cognitive counterpart of REM sleep (although dreams under NREM sleep may also occur), the long-lasting REM sleep suppression induced by ibogaine administration seems at first sight to be against its previously mentioned oneirogenic properties. However, this may not be the case, since a quantitative analysis of the EEG during W could provide evidence of an altered W pattern. For example, ibogaine-induced W may present a decrease in gamma band coherence, which is a well-known electrophysiological signature of REM sleep (Castro et al., 2013; Cavelli et al., 2015, 2017a). In other words, this pharmacologically-induced W state could have subtle electrophysiological traits of REM sleep that could explain the oneirogenic cognitive effect of the drug. With the aim of answering this question, a quantitative analysis of the EEG (power, coherence, co-modulation, entropy) is under process and will be reported in due course. In addition, this type of analysis would be helpful to compare the quality of W produced by ibogaine to traditional psychedelics.

Which are the underlying mechanisms of the W-promoting and REM sleep suppressive effects? Experiments in synaptosomes have shown that ibogaine inhibits serotonin re-uptake, which increases the synaptic levels of this neurotransmitter (Wells et al., 1999). In addition, Wei et al. (1998) showed that ibogaine elicited a large increase in serotonin levels (up to 25-fold in the Nucleus Accumbens, NAC and 10-fold in the striatum, STR) while noribogaine produced a moderate increase (up to eightfold in NAC and fivefold in STR) (Wei et al., 1998). In contrast, Baumann et al. (2001b) showed that noribogaine was more potent in increasing serotonin levels at the NAC than ibogaine, which correlates with the ability of both compounds to inhibit SERT (IC_50_ of 3.85 and 0.18 μM for ibogaine and noribogaine, respectively). These authors suggested that ibogaine and noribogaine are serotonin-reuptake inhibitors with a mechanism of action similar to fluoxetine. Serotonin increases W and suppress the generation of REM sleep (Oniani and Akhvlediani, 1988; Monti and Jantos, 2005). Hence, the ability of ibogaine and noribogaine to increase synaptic serotonin concentration could account for the increment in W time, as well as the long-lasting REM suppressive effect. The fact that most antidepressant drugs share this REM sleep suppressing effect (Palagini et al., 2012), insinuate that ibogaine might also have antidepressant properties as suggested by human reports (Mash et al., 2000).

Schneider and Sigg (1957) also proposed that the activation of the cholinergic pathways should be involved in the effects produced by ibogaine. In this regard, mesopontine and basal forebrain cholinergic neurons are involved in the generation and maintenance of W (Torterolo et al., 2016). Hence, it is likely that by modulation of these neurochemical systems ibogaine can promote W and suppress sleep. Nevertheless, because of its complex pharmacology, the interactions between other neurotransmitters, as well as the still unknown effects of ibogaine on REM sleep promoting neurons such as the melanin-concentrating hormone (MCH) containing neurons, should be taken into account when interpreting these results (Torterolo et al., 2011; Monti et al., 2013).

Another possibility is that the increase in W could be caused by some unspecific effect, such as irritation or pain. In this regard, we did not observe any behavior suggesting this kind of effect. In fact, it is interesting to consider that there is evidence of ibogaine as an anti-nociceptive agent (Olney, 1995, 1997). Moreover, both ibogaine and noribogaine enhance morphine anti-nociception (Bagal et al., 1996), and to our knowledge, there are no observational studies which reported pain or inflammation after ibogaine administration in humans.

When considering the results seen in each 2-h block, we hypothesized that the different effects seen along the entire recording could be attributed not just to ibogaine itself, but also to its principal metabolite, noribogaine. As mentioned before, according to previous reports using i.p. administration in rats, ibogaine concentration in blood rapidly decreases in the first hour (with a T-max of approximately 0.1 h), while noribogaine is detectable in blood up to 24 h after ibogaine administration (with a T-max of approximately 2.4 h) (Baumann et al., 2001b). Hence, the increased W found in the first 2-h block could be correlated to the peak concentration of ibogaine in addition to increasing amounts of noribogaine, while the extended REM suppression seen through the entire recording could be attributed to the long-lasting noribogaine. Further experiments are needed to confirm this hypothesis.

Regarding motor behavior, a higher total locomotor activity was found after *I*_20_ administration suggesting a more vigilant animal response. This stimulant effect is in accordance with previous findings that showed that 1 and 10 mg/kg i.v. doses promoted a dose-dependent increase in locomotor activity in rats (Baumann et al., 2001b). In contrast, *I*_40_ administration did not show a substantial increase in locomotor activity, producing serotonin syndrome-like behaviors such as tremor and flat body posture mainly during the first part of the recording session. It is likely that the appearance of these behaviors would explain, at least in part, the abnormal environment animal habituation response to the OF (**Figure 3A**), and reduced the ability to induce rearing behavior that was found after the administration of this dose (**Figure 3B**). Taken together, these results clearly indicate that *I*_40_ induces a kind of W that is different than the one produced by the lower dose. These results may resemble subjective experience of natives from Congo and Gabon who used low doses of *T. iboga* root bark as a powerful stimulant to combat fatigue and tiredness, while larger doses were chosen to produce visions in ritual settings (Schneider and Sigg, 1957; Pope, 1969).

The ibogaine induced tremor and flat body posture (**Figure 4A**), suggest a putative interaction with serotonin transmission. However, Baumann et al. (2001b) have postulated that a serotonergic mechanism may not be involved in the locomotor effects of ibogaine, since according to their results noribogaine (which is not tremorgenic in rats) is more potent in increasing serotonin levels than ibogaine. In this manner, they speculate that sigma or NMDA receptors might also explain these behaviors (since noribogaine has less affinity for this sites) (Baumann et al., 2001b). Regarding the induction of HSR, no changes were found for both ibogaine treatments (**Figure 4B**). As mentioned before, this behavior is exacerbated by the systemic administration of 5HT_2A_ receptor agonists (like classical hallucinogens). This constitutes a putative behavioral difference for animals treated with hallucinogens (LSD, DOI, etc.) versus ibogaine, which is considered a non-traditional psychedelic. Pharmacological data also favor this difference, while hallucinogens interact with the 5-HT_2A_ receptor in the nanomolar range, ibogaine affinity for this receptor is in the micromolar range (*K_i_* 4.8–92.5 μM depending the study) (Repke et al., 1994; Sweetnam et al., 1995; Helsley et al., 1998; Glick et al., 1999) or negligible (Deecher et al., 1992; Staley et al., 1996). At last, this difference is also supported by the differences between the subjective experiences in humans where ibogaine does not produce the typical interferences in thinking, identity distortions, and space–time alteration produced by the traditional psychedelics drugs (Naranjo, 1973). In summary, the sleep and motor effects induced by ibogaine, and its differences with classical hallucinogens, can only be explained by the interaction of ibogaine with several neurochemical systems.

## Conclusion and Future Perspectives

In this study, we observed that intraperitoneal administration of ibogaine in rats produced an increase in W, a decrease in SWS along with a robust suppression of REM sleep. Although these effects on sleep and W were observed for both doses of ibogaine (20 and 40 mg/kg), locomotor studies indicated differences in the behavioral outcomes for both treatments. While the *I*_20_ dose had a stimulant profile, the *I*_40_ dose generated an abnormal environment habituation where significant tremor and flat body posture were detected. Hence, given that ibogaine is considered an oneirogenic psychedelic, the next step would be to analyze the specific electroencephalographic characteristics of the pharmacologically induced W, such as the power spectrum and spectral coherence between different cortical areas. This kind of analysis could provide further insights of the cognitive activity induced by this drug.

## Ethics Statement

This study was carried out in accordance with the recommendations of the National Animal Care Law (No. 18611) and with the “Guide to the care and use of laboratory animals” (8th edition, National Academy Press, Washington DC, 2010). Furthermore, the Institutional Animal Care Committee (Facultad de Medicina – Universidad de la República; Instituto de Investigaciones Biológicas Clemente Estable) approved the experimental protocols. Adequate measures were taken to minimize pain, discomfort, or stress of the animals, and all efforts were made to use the minimal number of animals necessary to obtain reliable scientific data.

## Author Contributions

IC, GS, PT, and CS provided the financial support. IC, PT, and CS performed the experimental design. JG, JP, PR, MC, LB, AM, and MP performed the experimental procedures. JG, MC, JP, IC, PT, CS, and GS were involved in the analysis of the data. JG, MC, JP, GS, IC, PT, and CS were involved in the discussion and interpretation of the data. JG, IC, PT, and CS wrote the manuscript. All the authors participated in the critical revision of the manuscript, added important intellectual content, and approved the definitive version.

## Conflict of Interest Statement

The authors declare that the research was conducted in the absence of any commercial or financial relationships that could be construed as a potential conflict of interest.
