# Supplementary material for: Ibogaine Acute Administration in Rats Promotes Wakefulness, Long-Lasting REM Sleep Suppression, and a Distinctive Motor Profile
Source: Front Pharmacol. 2018 Apr 27;9:374. doi: 10.3389/fphar.2018.00374 (PMC5934978; doi:10.3389/fphar.2018.00374)
Supplement: Supplementary file 1 [file Presentation_1.pdf]

frontiers

Supplementary Material

Ibogaine Acute Administration in Rats Promotes Wakefulness, Long-Lasting REM Sleep Suppression, and a Distinctive Motor Profile

Joaquín Gonzalez[1], José Pedro Prieto[2], Paola Rodríguez[3], Matías Cavelli[1], Luciana Benedetto[1], Alejandra Mondino[1], Mariana Pazos[3], Gustavo Seoane[3], Ignacio Carrera[3]*, Cecilia Scorza[2]*, Pablo Torterolo[1]

* **Correspondence:** Cecilia Scorza (scorzacecilia@gmail.com), Ignacio Carrera (icarrera@fq.edu.uy)

1 Supplementary Data

1.1 Ibogaine-HCl

Nuclear Magnetic Resonance spectra were obtained in CD_3OD on a Bruker Avance DPX-400 instrument.

^{1}H NMR (400 MHz, CD_3OD) δ (ppm) =7.19 (d, *J* = 8.9 Hz, 1H), 6.97 (d, *J* = 2.4 Hz, 1H), 6.76 (dd, *J* = 8.8, 2.5 Hz, 1H), 3.87 (s, 3H), 3.70 (dt, *J*= 13.4, 4.2 Hz, 1H), 3.63 – 3.53 (m, 2H), 3.45 – 3.34 (m, 3H), 3.31 – 3.14 (m, 2H), 2.32 (ddt, 13.5, 12.1, 2.7 Hz, 1H), 2.19 – 2.09 (m, 2H), 2.06 (hept, *J* = 7.5 Hz, 1H), 1.74 – 1.65 (m, 3H), 1.46 – 1.34 (m, 1H), 1.03 (t, *J* = 7.3 Hz, 3H) **^{13}C NMR** (100 MHz, CD_3OD) δ(ppm) = 153.0, 139.1, 130.4, 128.5, 111.2, 111.1, 106.0, 99.5, 60.1, 56.0, 54.9, 50.5, 39.0, 35.1, 31.2, 28.8, 26.0, 23.9, 18,0, 10.5

Gas Chromatography analysis was carried out in a GC-MS Shimadzu QP 1100 EX instrument using the electron impact mode, 70 eV. Column HP-5MS (30m x 0.25mm x 0.25um) Temperature Program 200 ºC (Hold time, 2 minutes) to 300 ºC (Hold time, 5 minutes) with a rate of 10 ºC/min. Ibogaine purity was determined as 96.4%, and ibogamine and ibogaline were detected as trace impurities.

Retention Time (minutes)	% Area	Structure
10.521	2.05	Ibogamine
12.640	96.38	Ibogaine
13.383	0.34	Unkown
14.441	1.23	Ibogaline

2 Supplementary Figures and Tables

2.1 Supplementary Figures

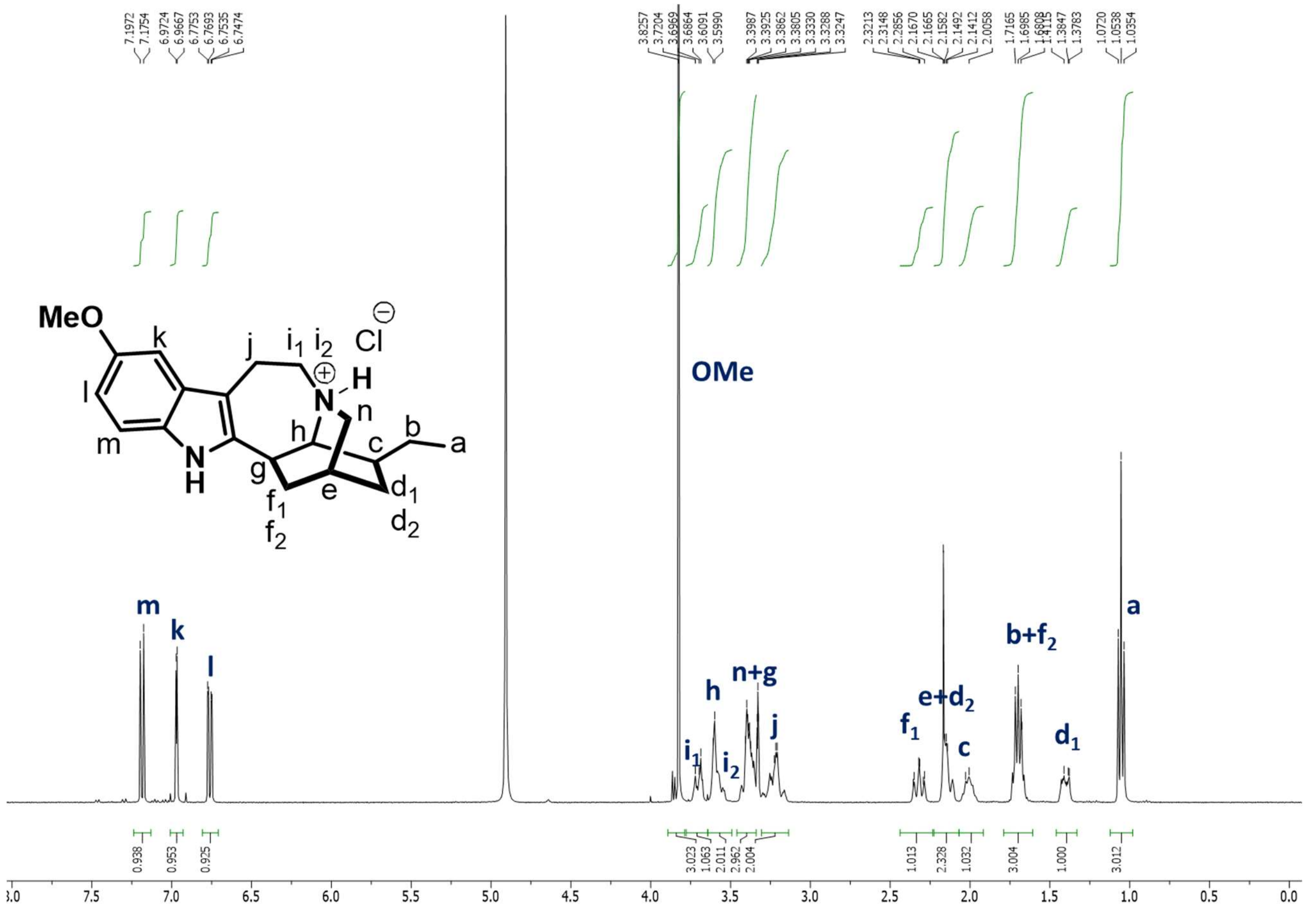

Supplementary Figure 1. Ibogaine-HCl ^{1}H NMR. Diastereotopic protons are labeled as x_{1}/x_{2}. Solvent residual peak for MeOH is found at 3.33 ppm, and H_2O at 4.90.

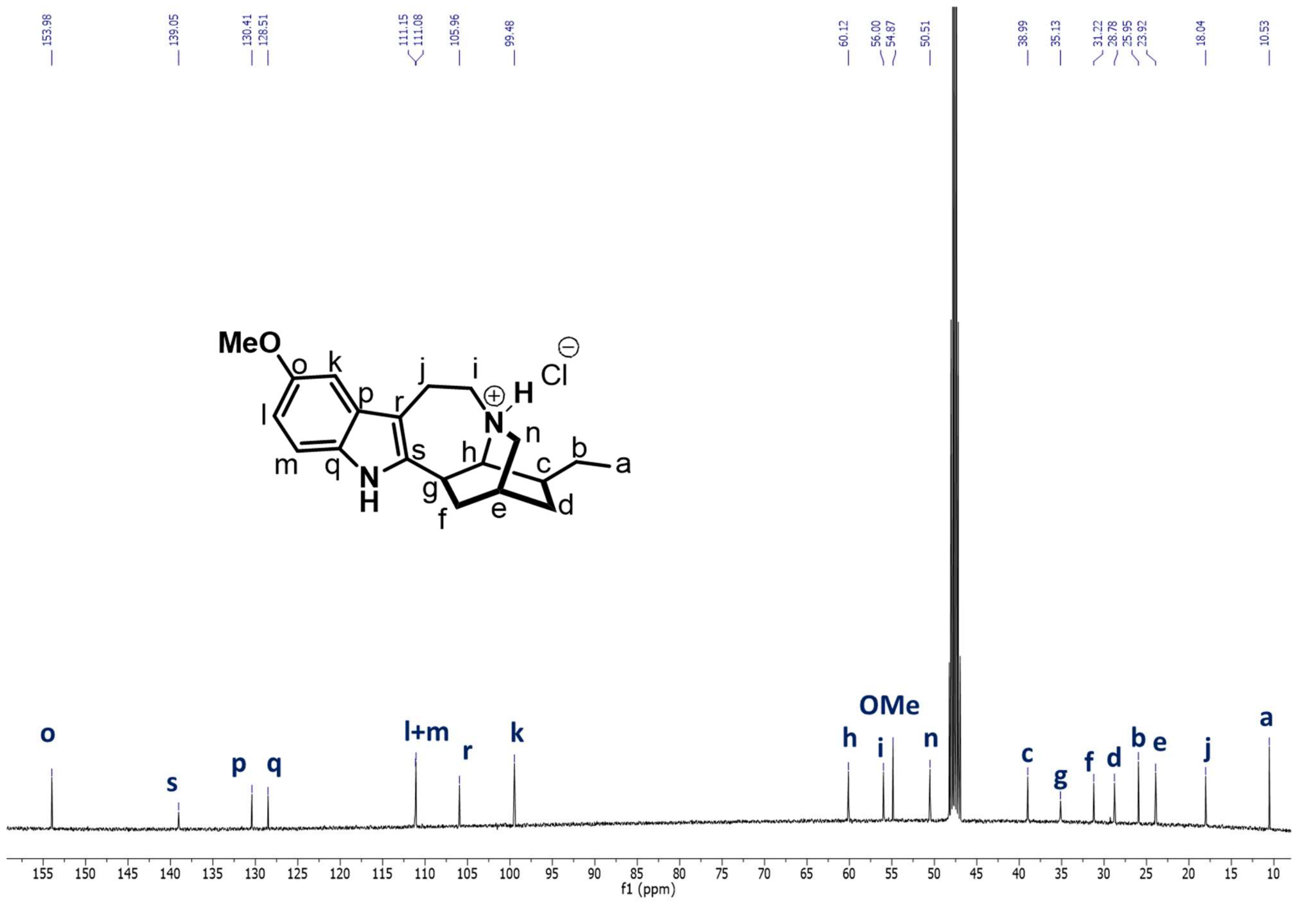

Supplementary Figure 2. Ibogaine-HCl ^{13}C NMR.

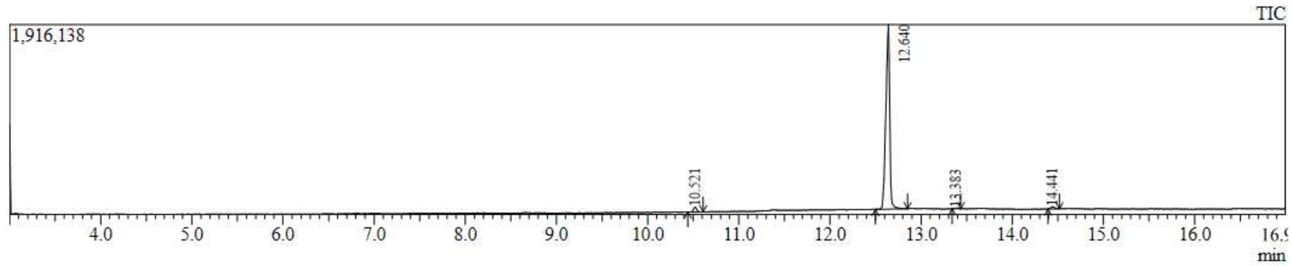

Supplementary Figure 3. Ibogaine-HCl GC-MS chromatogram
